# Motor Plans under Uncertainty Reflect a Trade-Off between Maximizing Reward and Success

**DOI:** 10.1523/ENEURO.0503-21.2022

**Published:** 2022-04-08

**Authors:** Aaron L. Wong, Audrey L. Green, Mitchell W. Isaacs

**Affiliations:** 1 Moss Rehabilitation Research Institute, Elkins Park, PA 19027; 2 Department of Neuroscience, Holy Family University, Philadelphia, PA 19114

**Keywords:** decision making, intermediate movements, motor planning, reward, subjective value, uncertainty

## Abstract

When faced with multiple potential movement options, individuals either reach directly to one of the options, or initiate a reach intermediate between the options. It remains unclear why people generate these two types of behaviors. Using the go-before-you-know task (commonly used to study behavior under choice uncertainty) in humans, we examined two key questions. First, do these two types of responses actually reflect distinct movement strategies? If so, the relative desirability (i.e., weighing the success likelihood vs the attainable reward) of the two target options would not need to be computed identically for direct and intermediate reaches. We showed that indeed, when reward and success likelihood differed between the two options, reach direction was preferentially biased toward different directions for direct versus intermediate reaches. Importantly, this suggests that the computation of subjective values depends on the choice of movement strategy. Second, what drives individual differences in how people respond under uncertainty? We found that risk/reward-seeking individuals tended to generate more intermediate reaches and were more responsive to changes in reward, suggesting these movements may reflect a strategy to maximize reward versus success. Together, these findings suggest that when faced with choice uncertainty, individuals adopt movement strategies consistent with their risk/reward attitude, preferentially biasing behavior toward exogenous rewards or endogenous success and consequently modulating the relative desirability of the available options.

## Significance Statement

When choosing between multiple options, individuals weigh the relative values of the options (i.e., how rewarding it is and how likely one is to successfully obtain that reward). Moreover, when the outcome is uncertain, individuals typically adopt one of two movement strategies: commit to an option immediately and reach directly for it, or hedge one’s bets by reaching intermediate between the options until more information can be obtained. Here we show that the relative values of the options are not fixed, but change depending on the selected movement strategy (direct or intermediate reaches). These strategies in turn are related to an individual’s tendency to be risk/reward-seeking. Thus, option values depend on how individuals choose to respond to uncertainty.

## Introduction

When selecting between multiple potential options, we plan our response by weighing all available options to maximize performance. When individuals must initiate their movement before the correct option can be identified, they respond in one of two distinct ways. First, individuals may commit to one of the options before movement onset, subsequently moving directly toward that option (i.e., a direct response). In contrast, individuals may generate a movement that initially lies partway between the available options (i.e., an intermediate response). One popular hypothesis for why intermediate movements are generated proposes that individuals prepare a motor plan to each of the potential options, and averaging between these plans (unintentional or otherwise) gives rise to intermediate movements (Cisek, 2007; Chapman et al., 2010; Gallivan et al., 2016, 2018; Enachescu et al., 2021). Under this averaging hypothesis there is no distinct motor plan to produce an intermediate response; instead, the intermediate “plan” is simply a weighted average of direct-response plans. In contrast, increasing evidence favors an alternative hypothesis that intermediate responses reflect a strategy to improve performance by delaying commitment to either option until more information can be attained, minimizing the expected cost of generating motor corrections (Hudson et al., 2007; Nashed et al., 2017; Wong and Haith, 2017; Dekleva et al., 2018; Alhussein and Smith, 2021; Onagawa and Kudo, 2021). Under this alternative hypothesis, an intermediate movement is deliberately planned as a strategic response to uncertainty.

Aside from deciding whether to generate a direct or an intermediate response, another important factor governing choices in the face of uncertain options is to weigh the relative desirability of the two options. In particular, prior research has demonstrated that individuals consider both the potential rewards available as well as the likelihood with which those rewards will be successfully attained (for review, see Wolpert and Landy, 2012; Shadmehr et al., 2019). By reward, we mean an exogenous signal (in our case, money) conferred by the experimenter indicating correct behavior; by success likelihood, we mean the probability that the chosen action will be correct and result in an endogenous satisfaction signal. Both when choices are discrete (e.g., direct reaches or saccades) as well as when intermediate movements are generated, movements tend to be biased toward the more rewarding (Sugrue et al., 2004; Liston and Stone, 2008; Chapman et al., 2015) or likely (Liston and Stone, 2008; Chapman et al., 2010) option, reflecting an effort to maximize reward or success, respectively. Note, that reward and success likelihood can be manipulated independently, and can even be set in opposition. When both reward and success likelihood are unequal between the two options, behavior is ideally determined by comparing the expected values (i.e., reward times success likelihood) of the options (Teichert and Ferrera, 2010; Johnson and Ratcliff, 2014).

However, neuroeconomics reveals that individuals do not evaluate options according to their objective expected value, but according to their subjective value (utility; Kahneman and Tversky, 1979; Mongin, 1998; Glimcher and Rustichini, 2004). That is, individuals might preferentially weigh success likelihood or reward more heavily in their decisions, leading to choice biases even in cases where the options have equivalent expected values. Our first goal was therefore to examine whether reward and success likelihood contributed equally when determining the relative subjective values of the two reach options, as determined by a bias in reach direction when the objective value of the two targets was matched, and whether this bias was analogous for both direct and intermediate reaches.

Prior studies examining intermediate reaches have all focused their analyses at the group level. What is frequently not discussed is the fact that individuals often differ greatly in how they respond in these tasks, although every participant is presented with the exact same set of options. While some individuals preferentially generate intermediate responses, others produce only direct responses, and still others produce a mixture of both behaviors. No clear rationale has been proposed as to why, given the exact same set of options on every trial, different individuals will respond with different proportions of these two types of behaviors. Our second goal, therefore, was to examine whether we could explain differences in the degree to which individuals preferentially generate direct or intermediate movements.

To answer these questions, we asked participants to perform a go-before-you-know task in which they were required to initiate reaches toward targets without knowing which option was the correct choice. One target was more rewarding while the other was more likely to result in success. We evaluated the degree to which reward and success likelihood biased behavior for both direct and intermediate reaches. We also examined whether individual risk/reward attitude could explain behavior at the individual-subjects level. Together, these findings shed light on how individuals decide to move under uncertainty.

## Materials and Methods

Sixty-nine right-handed adult neurotypical individuals were recruited for this study. Of those individuals, 24 participated in experiment 1 (age 18–40, average 26.0 ± 5.1; 17 female), 17 participated in experiment 2 (age 22–34, average 24.1 ± 2.8; 16 female), and 28 participated in experiment 3 (age 21–33, average 24.8 ± 2.4 years; 22 female). Of these 28 individuals in experiment 3, 19 participants reliably generated both direct and intermediate reaches in every condition (i.e., across the varying reward and frequency ratios) such that the relative weighting of frequency and reward could be estimated; the analyses here thus reflect the data from these 19 individuals. In contrast, in experiment 1, only 13 out of 24 participants reliably made both direct and intermediate reaches (i.e., at least 5% of each reach type) in every block, and in experiment 2, only 8 out of 17 participants reliably made both direct and intermediate reaches. Hence, data from these two experiments were analyzed at the population level. Sample sizes were chosen to be comparable to or greater than previous studies using a similar task (Chapman et al., 2010; Wong and Haith, 2017). All participants were naive to the purposes of this study, and provided written informed consent. Experimental methods were approved by the Albert Einstein Healthcare Network Institutional Review Board and participants were compensated for their participation as a fixed hourly payment ($12/h) plus the potential to earn an additional bonus (up to $6/h) based on performance in the task.

Participants made planar reaching movements while seated in a Kinarm Exoskeleton Lab (Kinarm). Hands, forearms, and upper arms were supported by the robot in troughs appropriately sized to each participant’s arms, and the linkage lengths of the robot were adjusted to match limb segment lengths for each participant to allow smooth motion at the elbow and shoulder. Vision of the arm was obstructed by a horizontal mirror, through which participants viewed a cursor (1-cm diameter) representing the position of the index finger and targets (2-cm diameter) displayed by an LCD monitor (60 Hz) in a veridical horizontal plane. Movement of the arm was recorded at 1000 Hz.

### Experiment 1

**Figure 1.**
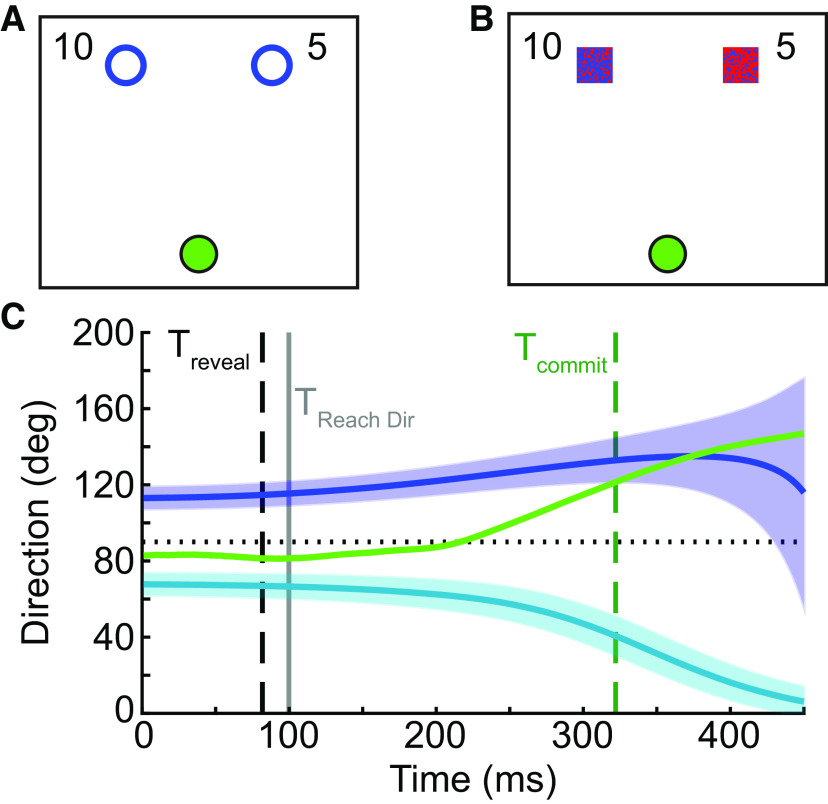
Methods for experiments 1 and 2. Participants completed a go-before-you-know task in which they observed two targets and were required to begin moving; only after they began their movement would the true target would be revealed. ***A***, In experiment 1, one of the two target options could be correct more frequently, and the other target could result in more reward. Reward offers appeared explicitly on screen (e.g., 10 vs 5 cents, or a reward ratio of 2:1), the two targets were otherwise identical, and participants had to learn the frequency manipulation through experience. ***B***, In experiment 2, target probability was modulated on each trial instead of blockwise frequency; target probability on the current trial was indicated by the color of the target (the degree of redness reflects the probability of being correct, e.g., 80% vs 20%). ***C***, At each time point, the instantaneous hand direction (green) was computed relative to the direction required to be moving toward one of the two targets (blue, teal; the thick line represents the center of the target, and the shaded region represents the target width). The time at which the hand was heading directly toward one of the two target options (T_commit_, dashed green) was compared with the time when the correct target was revealed (T_reveal_, dashed black). If T_commit_ occurred before T_reveal_ the movement was classified as direct, otherwise it was classified as intermediate. This was estimated independently of when the initial reach direction was measured, which was always 100 ms into the reach (T_ReachDir_, gray).

Participants were asked to complete a “go before you know” task (Chapman et al., 2010; Wong and Haith, 2017) in which after a brief random delay (900 ± 600 ms) two potential targets (rings, 2-cm diameter) were presented 45° apart, 20 cm from a central starting position at the beginning of each trial (Fig. 1*A*). Participants were free to move any time after the targets appeared, and had 2500 ms from the time of target appearance to complete their reach by shooting through the target. Once participants began their movement (moved 4 cm from the starting position or exceeded a hand velocity of 0.1 m/s), the correct target (i.e., the one that had to be hit to earn a reward) was revealed by becoming solid in color and the distractor target disappeared. Participants were rewarded for hitting the correct target while maintaining a minimum hand velocity (0.3 m/s) without exceeding the vertical target distance from the starting position before hitting the target. Successful target interception resulted in a reward, the amount of which was determined according to an initial utility assay (see below for details). If participants moved too slowly, a “too slow” message was displayed at the end of the trial and participants did not earn a reward for that trial.

Each target was associated with a reward and a success likelihood, which varied per block. Monetary rewards were set as ratios of 1:1, 2:1, 5:1, and 10:1, and the reward amount assigned to each target in cents was displayed to the participant on every trial (Fig. 1*A*). Each target was also associated with a success likelihood according to the relative frequency with which that target was correct in each block; frequency ratios were 1:1, 2:1, 5:1, and 10:1. Participants were not informed that the targets would vary in frequency, but could learn these frequencies through experience. In all cases, the more rewarding target was always the less frequent target. Therefore, within a single block, the more rewarding target always appeared on the same side; the more-rewarding (lower-frequency) target side was counterbalanced across blocks and participants. Since target frequencies were learned by experience, participants always completed all blocks in the same order: increasing reward ratios at a frequency ratio of 1:1, followed by increasing reward ratios at a frequency of 2:1, etc. Because target frequencies varied between blocks, blocks consisted of 30–44 trials each. At the end of the session, participants were paid a fraction of the total rewards earned throughout these blocks, and thus were encouraged to treat all trials as if they would actually earn the reward offered for hitting the correct target.

**Figure 2.**
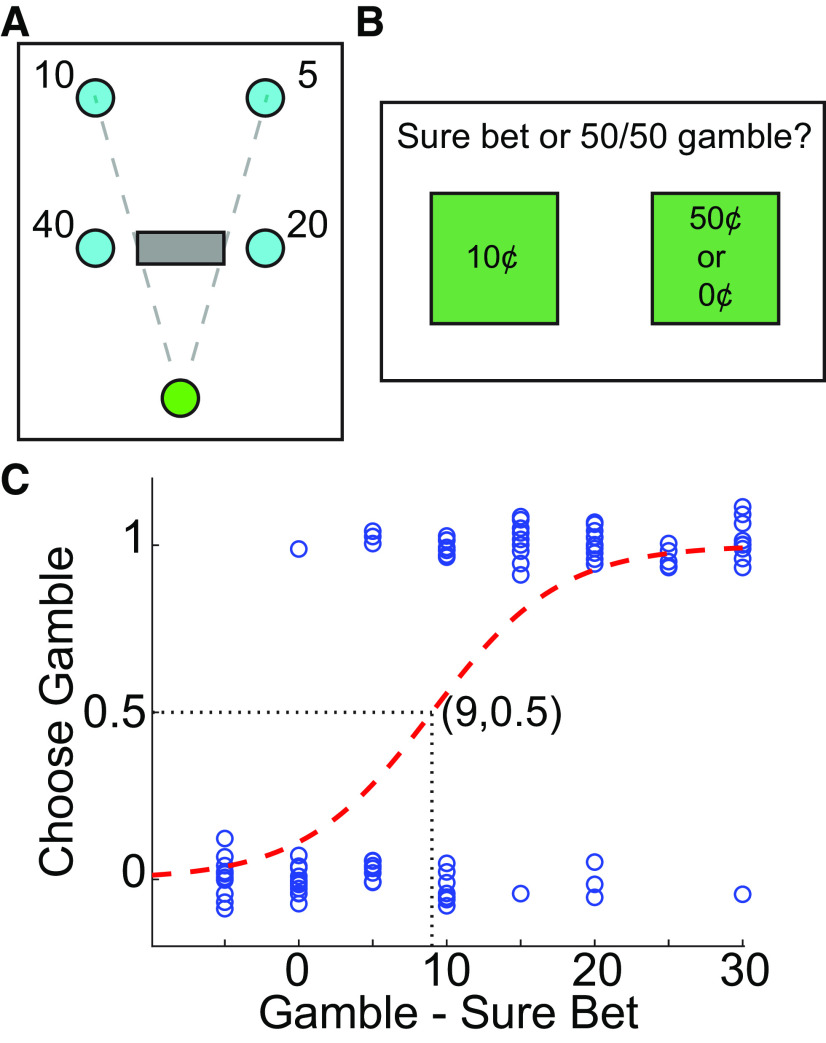
Methods for experiment 3. ***A***, Participants completed a modified go-before-you-know task in which intermediate reaches were explicitly indicated. Specifically, starting from a home position (green), participants could reach directly to one of two near targets with no knowledge of which target was correct until after hitting the target, or they could pass through a rectangle that would cause both near targets and one of the far targets to disappear, revealing the correct far target. Both leftward or both rightward targets were “correct” on a given trial, and were assigned the same ratio of reward and likelihood. The reward offered for the far targets was a proportion of the reward available on the near targets; varying this proportion changed the relative desirability of making direct or intermediate reaches. The dashed lines were not visible to participants, but have been drawn to indicate the width of the “intermediate” rectangle relative to the far targets. ***B***, Participants also completed a utility test in which on each trial they indicated with a button press whether they preferred a sure bet or a 50/50 gamble. ***C***, Psychometric curves were fit to the choices made in the utility test, and the indifference point was estimated. Rightward shifts of the indifference point reflect greater risk aversion; leftward shifts reflect greater risk/reward-seeking tendency. Vertical jitter of data points was added for visualization purposes.

Before this session, participants completed a utility test (Fig. 2*B*). Since not all people value fixed monetary amounts to the same extent, in experiments 1 and 2 an effort was made to equate reward utility across participants such that all individuals would be similarly motivated by the individual target rewards. To do so, participants completed a pretest in which on every trial they were presented with two options, a fixed monetary amount (“sure bet,” e.g., 10 cents) and a 50–50 gamble (“gamble,” e.g., $1.00 or 0 cents). Participants had to choose which they would prefer; to have the fixed amount of money or take a gamble and win either of the two amounts depending on the outcome of a fair coin flip. Participants were not informed of the outcomes of their choices until the end of the block, where they saw a total score reflecting the sum of all their choices. Hence, success or failure of the gamble option did not impact the decision on the next trial. Participants received a fraction of the total earned as part of their final monetary compensation; thus, they were instructed to treat each trial as if they would actually earn the outcome of that trial. Immediately following this test, we estimated the likelihood of choosing a fixed amount versus the expected outcome of the gamble for each presented pair of options, and using a linear fit, estimated the fixed value for which the participant was equally likely to choose that fixed value versus a gamble with an expected outcome of 10 cents (i.e., the subjective point of equivalence). If the estimated value was <5 or >20 cents, we adjusted the value to be within this range to avoid offering excessively large or small reward values to participants. This monetary value was then set to be the base reward value in the go-before-you-know experiment above from which all the reward offers were computed (as multiples of the base reward value). The exact same Utility test was administered for each participant at the start of all three experiments. Code to run this experiment is available on our lab GitHub page (https://github.com/CML-lab/KINARM_GBYK_RwdFreq and https://github.com/CML-lab/Utility_Task_cpp).

### Experiment 2

Experiment 2 was identical to experiment 1 except that instead of varying the frequency associated with each target, we varied the target probability on a trial-by-trial basis (such that there was an equal long-run success likelihood of the right or left target being correct and the blockwise expected value of the two targets was equal, but on a particular trial each target was explicitly associated with a particular probability of being correct). Participants were informed of these probability ratios based on the appearance of the target; targets consisted of 2-cm squares divided into a checkerboard pattern, where the ratio of randomly chosen red to blue pixels in the target represented the target probability of being correct (Fig. 1*B*). Target probabilities could be 50:50, 70:30, or 80:20; 20 trials at each probability ratio were randomly intermixed within a single block for a total of 60 trials each. Reward ratios could be either 1:1, 2:1, or 5:1; each block contained a single reward ratio for every trial, with all participants experiencing reward ratios that increased throughout the experiment to minimize any potential biases because of moving from high-reward-ratio to low-reward-ratio trials. To ensure participants had sufficient time to observe the stimuli (probabilities and rewards available), the targets and reward values appeared 900 ± 300 ms before a go tone indicating that the reaching movement could begin. As above, a Utility test was performed and the result was used to set the base reward value on an individual-subject basis. Code to run this experiment is available on our lab GitHub page (https://github.com/CML-lab/KINARM_GBYK_RwdProb).

### Experiment 3

Unlike the first two experiments, experiment 3 presented individuals with five targets at the start of each trial: a near pair of circular targets 90° apart and 10 cm from the start position (7.07 cm in depth), a rectangular box spanning 46° across the vertical midline and 7.07 cm from the start position in depth (i.e., in line horizontally with the near targets), and a far pair of targets located 45° apart and 15 cm from the start position (13.86 cm in depth; Fig. 2*A*). Thus, in this experiment, participants were presented with two explicit choices; they could either directly reach for one of the two targets closest to the start position, or they could pass through the rectangle on their way to the far targets (representing an explicit “intermediate” option). If participants reached toward one of the near targets, the targets remained on screen unchanged until one was hit, after which they received feedback on whether they hit the correct or incorrect target. In contrast, when participants passed through the rectangle, the closest pair of targets vanished as well as the incorrect far target, leaving only the correct far target visible toward which participants could steer their reach. To give participants time to assess the greater complexity of the visual display, participants were required to view the screen for at least 2 s before a tone sounded to indicate participants could begin moving.

The two targets on a given side of the midline were yoked together, such that the “correct” target was either the two targets on the right or the two targets on the left. That is, participants primarily had to choose whether to aim rightward or leftward. Participants were informed of this relationship between the targets. As in experiment 1, correct targets appeared with differing frequencies in each block (with fixed frequency ratios of 1:1, 2:1, and 4:1); the side of the more frequent target differed across blocks within an individual, and was counterbalanced across participants. In addition, the reward ratio of the two near targets was the same as the reward ratio of the two far targets, in ratios of 1:1, 2:1, and 4:1. However, the pair of targets closer to the start position were worth anywhere from one to four times as much as the two targets farther from the start position. Thus, when the near targets were worth four times the value of the far targets, a direct reach strategy was implicitly encouraged; when the near targets were worth the same value as the far targets, an intermediate reach strategy was implicitly encouraged. The reward values of the four targets were explicitly written on the screen. Reward values (in cents) were fixed for all participants such that each participant saw the exact same reward values throughout the experiment (i.e., reward values were not adjusted in response to risk/reward attitude), with a base value of 5 cents (e.g., greatest reward offered was 5 × 4 × 4 = 80 cents). The more rewarding side was counterbalanced across blocks such that participants could not predict from one block to the next which side would offer more reward or be more frequently correct. The overall result of this experimental design was to encourage individuals to generate both intermediate and direct reaches regardless of the exact target reward and frequency, moreover, we were able to explicitly identify reaches that were “direct” versus “intermediate” without resorting to model fitting or assumptions in the analysis (see below). A Utility test was conducted (Fig. 2*B*), but the outcome of this test was not used to set the reward values during the session; instead, this test was used to estimate risk/reward sensitivity (see below). Code to run this experiment is available on our lab GitHub page (https://github.com/CML-lab/KINARM_GBYK_DiscreteChoice).

### Data analysis

Reaches were analyzed offline using programs written in MATLAB (The MathWorks). Data and analysis code are available at https://osf.io/jn79v/. Movement onset was identified according to a velocity criterion (tangential velocity >0.05 m/s in experiments 1 and 2, 0.02 m/s in experiment 3), and verified by visual inspection. Since experiment 3 did not require *post hoc* classification of reaches as direct or intermediate, we allowed our algorithm to be more liberal in identifying movement onset as a time earlier during the reach. Regardless of the method used to identify movement onset, the initial reach direction was determined as the direction of the velocity vector 100 ms after movement onset. In experiment 1, reaches were excluded from further analysis if the initial reach direction was >45° away from the midline or the reach was not completed in 1000 ms from target appearance; this led to a median of 6.8% reaches being removed for each participant. In experiment 2, reaches were excluded if the initial reach direction was >45° away from the midline or the reach was not completed in 650 ms after the go tone (unlike experiment 1, during this experiment, we restricted participant’s movement time rather than the total time from target appearance); this led to a median of 11.4% of reaches removed. For experiment 3, reaches were excluded if they were >75° away from the midline (to account for potential avoidance of the center rectangle by reaching even farther from the midline than strictly necessary), the reach was not completed in 2500 ms (to account for longer movement times on intermediate reaches), or if the trial was incomplete; this led to a median of 10.5% of reaches removed.

In experiments 1 and 2, reaches were classified as “direct” or “intermediate” according to a time criterion. At each time point (i.e., data sample) during the reach from movement onset to the time the hand stopped moving, we calculated for each target the instantaneous target direction as the direction in which the hand would need to move, given its current position, to be heading directly toward the target (also accounting for the width of the target; see Fig. 1*C*). We also computed the instantaneous reach direction of the hand at each time point as the direction of the velocity vector (i.e., the tangent of the direction vector) at that time. By combining these two calculations, we were able to identify the time at which individuals first committed to reaching toward one of the two targets (T_commit_) as the time when the reach direction was equal to the instantaneous target direction. We noted whether T_commit_ occurred before or after the time that the true target was revealed (plus an additional 60 ms to account for delays between when the command was sent to modify the display and when individuals might actually perceive the display changing; T_reveal_). If T_commit_ occurred before T_reveal_, we considered this to be a “direct” reach; otherwise, the reach was classified as “intermediate.” Note that because of trial-to-trial variability in instantaneous hand velocity during the reach, this classification approach had no relationship to when the initial reach direction was determined, which was always measured a fixed 100 ms into the reach.

For experiment 3, reaches were explicitly identified as direct or intermediate depending on which target the reach first intercepted. Whenever a reach intercepted the rectangle, it was classified as intermediate, otherwise it was considered a direct reach. Because of the way the task was programmed, it was not possible for individuals to intercept both the rectangle and one of the near targets; hitting the rectangle caused the near targets to disappear leaving only the far targets as viable options, while hitting one of the near targets (or missing both near targets and the rectangle) caused the trial to end.

Classification of reaches (whether by a temporal criterion or direct examination) resulted in two reach-direction distributions: a bimodal distribution of reaches aimed directly to the two targets, and a unimodal distribution of reaches aimed intermediate between the two targets. For experiments 1 and 2, because of idiosyncratic behavior across individuals, reach directions across all individuals were pooled before analysis. Reach-direction distributions were analyzed separately by fitting a bimodal or unimodal Gaussian using the built-in fitgmdist and the fitdist functions in MATLAB, respectively. Variance associated with the fit parameters was estimated using a bootstrap approach in which Gaussians were fit to a randomly selected subset of 75% of the participants, and this subsampling was repeated 1000 times to estimate distributions for each of the fit parameters. Iterations were removed when one or more fits did not return valid parameters, or when by random chance insufficient numbers of direct or intermediate trials were available to fit. Of particular interest were the ratio of the two modes of the bimodal distribution (*D_choice_*), which reflects the relative frequency with which individuals chose the higher-rewarded but less-likely target on direct reaches, and the shift of the mean of the unimodal distribution away from zero (*I_bias_*), which reflects a bias in the initial reach direction toward the higher-rewarded but less-likely target for intermediate reaches.

Separately for direct and intermediate reaches, the effect of reward and success likelihood on reach-direction biases were estimated by fitting linear regressions to the parameter estimates (*D_choice_* or *I_bias_*, respectively) when the success likelihood or reward ratio of the two targets was 1:1, respectively. These regressions estimate how reward or success likelihood independently modulated reach direction. These regressions were used to estimate the expected *D_choice_* or *I_bias_* values for the cases when both reward and success likelihood were different between the two targets. In general, the *D_choice_* or *I_bias_* values calculated from an equal weighting (average) of reward and success likelihood effects were not observed to fit the actual data well (based on visual inspection). Thus, to identify the best relative weighting of reward and success likelihood information that could explain the behavioral data, we searched for the weighted sum of independent reward and success likelihood effects that best predicted the bootstrapped *D_choice_* or *I_bias_* values for each bootstrap iteration. These weights represented the relative contributions of reward and success likelihood information influencing reach direction, for direct or intermediate reaches, respectively. In all cases, values are reported as mean ± SD, unless otherwise noted to be reporting the SEM. Weight distributions were compared with 0.5 (equal weighting) using *t* tests, and to each other using a Kolmogorov–Smirnov test.

For experiment 3, because the task was designed to encourage both direct and intermediate reaches regardless of the combination of reward and success likelihood, data were analyzed at the individual-participant level in a within-subjects analysis. For direct reaches, we computed *D_choice_* as the proportion of reaches aimed at the more rewarding target over the total number of successfully completed reaches. For intermediate reaches, *I_bias_* was estimated as the mean reach direction on intermediate reaches. As in experiments 1 and 2, we used regressions to estimate the influence of reward or success likelihood alone on *D_choice_* or *I_bias_* when only one factor was changing; then we computed the weighted sum of the influence of reward and success likelihood that best explained the data in the cases when reward and success likelihood for the two targets were different. If reward and success likelihood exerted the same influence on direct and intermediate reaches, we would expect the relative reward and success likelihood weights to be similar for these two types of reaches. We compared the similarity of these weights for direct and intermediate reaches using a paired *t* test and examined their correlation using linear regression.

The proportion of intermediate reaches generated by each individual was calculated by counting the fraction of intermediate reaches produced in each condition, then taking the average. The degree to which the relative reward values of the near to far targets modulated the proportion of intermediate reaches was measured by finding, for a given ratio of near-target reward to far-target reward within each condition, the difference in the proportion of intermediate versus direct reaches. Then a regression was fit to examine the relationship between the near-to-far reward ratio and the difference in intermediate to direct reaches across all conditions. The slope of this regression was taken as an estimate of an individual’s sensitivity to the near-to-far reward ratio manipulation: steeper slopes (more positive values) indicated individuals who produced more direct compared with intermediate reaches when the near targets were more rewarding than the far targets, compared with when the near and far targets were equally rewarding.

Risk/reward attitude was examined in experiment 3 using data from the Utility test. For each participant, the choice (coded as 1 if choosing the gamble and 0 if choosing the sure bet) was plotted against the reward difference (the expected value of the gamble minus the value of the sure bet) on that trial (Fig. 2*C*). Then, a psychometric curve was fit to the data, and the indifference point was estimated as the point where the curve crossed 0.5 (i.e., an equal chance of choosing the gamble vs the sure bet). Larger indifference points reflect a more risk-averse (requiring a larger difference between the expected value of the gamble and the value of the sure bet to choose the gamble) and less reward-seeking (favoring the sure bet despite the greater potential reward to be earned by choosing the gamble) attitude. We examined the correlation between the proportion of intermediate reaches or the near-to-far reward sensitivity and individual risk/reward attitude.

Finally, reach accuracy was examined by measuring endpoint error, or the minimum absolute Euclidian distance between the correct target and the hand. Since reaches that were directed toward the incorrect target would skew the estimate of reach accuracy, all reaches made to the incorrect target (determined as trials when the endpoint error to the incorrect target was smaller than the endpoint error to the correct target) were first identified as target choice errors; then, the remaining reaches were classified as hits (the hand cursor intersected the target, or when the hand was ≤1.5 cm from the target center) or motor errors. We computed the proportion of total motor errors and choice errors separately for both intermediate and direct reaches, and asked whether the difference in the proportion of motor errors and choice errors were related to either the proportion of intermediate movements that people generated, or to the relative success-likelihood/reward weighting for direct or intermediate reaches, by fitting linear models in R using the lm() function.

## Results

### Frequency and reward bias behavior to different extents

**Figure 3.**
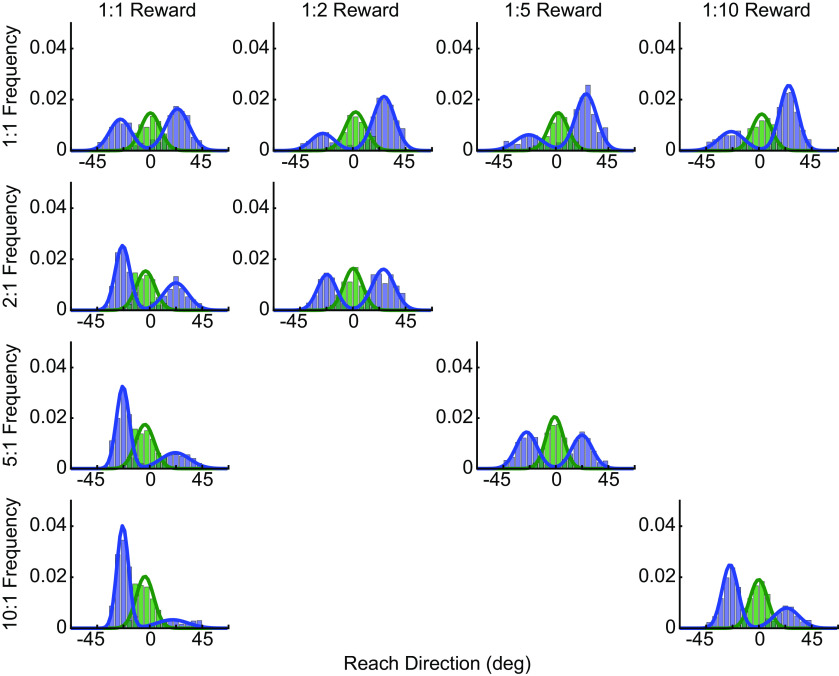
Reach-direction histograms for experiment 1. Initial reach direction for direct (blue bars) and intermediate (green bars) reaches (data pooled across all participants), with varying reward and frequency ratios. In all cases, the data have been aligned such that reaches to the more rewarded target are in the positive direction (rightward), and reaches to the more frequent target are in the negative direction (leftward). As the relative reward ratio increases (left to right), reaches are biased toward the more rewarded target. Likewise, as the relative frequency ratio increases (top to bottom), reaches are biased toward the more likely target. Fits reflect the average distribution parameters from bootstrapped fits to subsets of the pooled group distributions.

In experiment 1, participants completed a go-before-you-know task in which participants were presented with two potential targets and were required to initiate a reach before the correct target was revealed (Chapman et al., 2010; Wong and Haith, 2017). Each target was assigned a particular frequency (success likelihood) and reward value (Fig. 1*A*), with the exact frequency or reward varying across blocks. In response to these choice options, participants as a group generated a mixture of direct and intermediate reaches in all conditions. Gaussian distributions (bimodal for direct reaches and unimodal for intermediate reaches) were fit to the data to estimate reaching biases exhibited in each condition (Fig. 3). Because not every participant generated both direct and intermediate reaches in every condition, we used bootstrapping to estimate the relationship between the biases induced by varying reward and frequency for direct versus intermediate reaches (see Materials and Methods).

**Figure 4.**
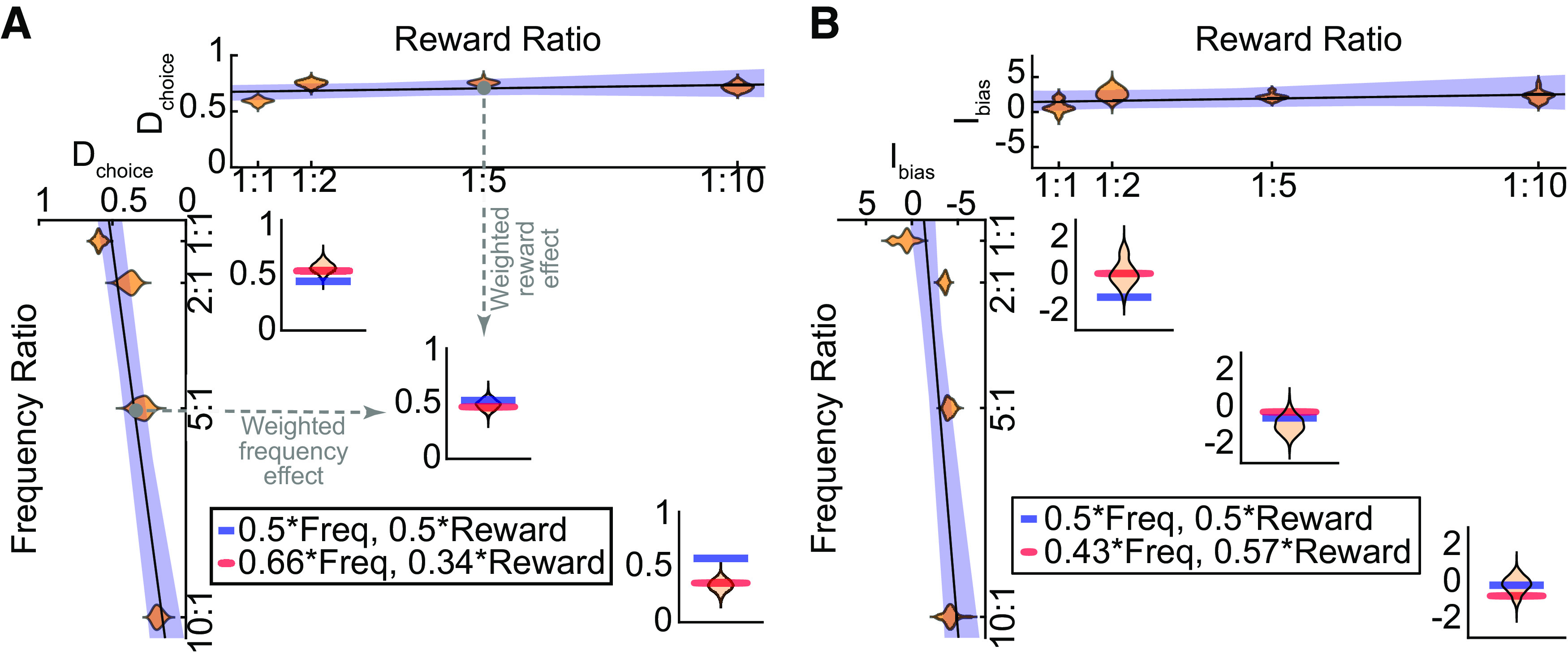
Estimation of biases in experiment 1. For direct reaches (***A***) and intermediate reaches (***B***), the effect of changing reward (top) or frequency (left side) alone was fit by a linear regression. These regressions were then used to predict the reach direction bias observed when both reward and frequency were changing (3 small panels; dashed arrows illustrate the estimation for one condition). Weighting the influence of frequency and reward unequally best explained the data (compare the red lines reflecting the best weighting of frequency and reward to the blue lines reflecting an equal weighting of frequency and reward). These weights indicated a much stronger influence of frequency compared with reward for direct reaches, but these factors were weighted differently for intermediate reaches.

In general, participants changed their reach biases as the relative frequencies and rewards of the two targets were modulated. For direct reaches, if the frequency of the two targets was equal (Fig. 4*A*, top panel), participants tended to make proportionally more reaches toward the target with the larger reward. This effect increased as the reward ratio of the two targets increased (slope = 0.01 ± 0.0038 SD). Likewise, if the two targets were rewarded equally (Fig. 4*A*, left panel), participants tended to make proportionally more reaches toward the target that was more frequently revealed to be the correct target, and this effect increased with the frequency ratio (slope = −0.04 ± 0.0041 SD). For intermediate reaches (Fig. 4*B*), a similar trend emerged although it was more difficult to detect by eye. Under conditions when the frequency of the two targets was equal (Fig. 4*B*, top panel), participants tended to exhibit initial reach directions that became increasingly biased toward the more rewarding target as the reward ratio increased (slope = 0.12 ± 0.10). When the rewards assigned to the two targets were equal (Fig. 4*B*, left panel), participants tended to reach in a direction that was biased toward the more frequent target particularly as the frequency ratio increased (slope = −0.37 ± 0.082).

Based on these effects of frequency and reward alone, we predicted how reaches would be biased when the effects of reward and frequency opposed each other. Specifically, the regressions above provided estimates of the relationship between frequency ratio and reach bias, and the relationship between reward ratio and reach bias. We then asked whether a (weighted) linear combination of these effects could explain the observed bias when frequency and reward both varied (see Materials and Methods). We found that rather than weighting frequency and reward equally, we could explain the observed biases best when the relative influences (weightings) of reward and frequency were allowed to be unequal (where the reward weight equaled one minus the frequency weight; Fig. 4*A*,*B*). For direct reaches, the best predictions were obtained if the influence of frequency on reach-direction bias was nearly twice that of reward (weight of frequency, 0.66 ± 0.11 SD; significantly different from 0.5, *t*_(999)_ = 44.90, *p *=* *8.21e-242).

**Figure 5.**
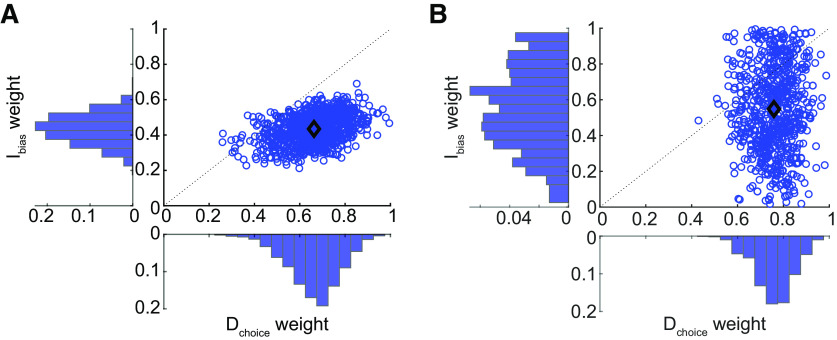
Comparison of likelihood-reward weight estimates for direct versus intermediate reaches, for experiments 1 and 2. ***A***, The relative weighting of frequency for direct (D_choice_ weight) and intermediate (I_bias_ weight) reaches, and their relationship (blue circles), is plotted for experiment 1. In this experiment, on average (black diamond), frequency tended to be weighed more heavily than reward for direct reaches, but the opposite weighting was observed for intermediate reaches. Such findings are consistent with an alternative analysis using a neuroeconomics-based approach (see Extended Data Fig. 5-1). ***B***, The same weight distributions and relationships are plotted for experiment 2 (see supporting data figures for experiment 2 in Extended Data Figs. 5-2, 5-3). Although there was much greater variability of probability-reward weight estimates for intermediate reaches in this experiment, on average weighting effects in experiment 2 were analogous to those observed in experiment 1 (compare the black diamond in panels ***A***, ***B***).

In contrast, for intermediate reaches, we observed the best predictions when reward was weighted slightly more than frequency [weight of frequency, 0.43 ± 0.08 (SD); significantly different from 0.5, *t*_(999)_ = −25.88, *p *=* *1.93e-113]. Overall, the weighting of frequency over reward was significantly greater for direct versus intermediate movements (weight difference = 0.23 ± 0.11 SD; paired *t* test, *t*_(999)_ = 64.47, *p *=* *0.00; Kolmogorov–Smirnov test, *p *=* *1.99e-272; Fig. 5*A*). These results agree with a second approach borrowed from neuroeconomics in which we assumed that the bias arose from a comparison of the relative subjective values of the two targets (see Extended Data Fig. 5-1). Such an analysis revealed that reward information was discounted more when estimating subjective value for direct reaches compared with intermediate reaches, consistent with the increased weighting of reward for intermediate reaches as observed above.

10.1523/ENEURO.0503-21.2022.f5-1 Extended Data Figure 5-1 Subjective valuation fits for experiment 1. In addition to estimating the relative weighting of reward and likelihood on observed reach biases, we also examined the relationship between the observed reach biases and the relative subjective values of the targets in conditions when the rewards and likelihoods of the two targets were unequal. This relationship was examined both for direct reaches (***A***) and intermediate reaches (***B***). In both cases, the left panels show the distribution of power law fits to the relationship between reward ratio and the measured bias divided by the frequency ratio of the two targets. The right panel shows a histogram of the estimated value of the exponent (α) applied to the reward ratio; larger α values reflect less discounted reward relative to likelihood in determining the relative subjective values of the two targets.
The analysis presented in Extended Data Figure 5-1 is based on typical neuroeconomic analyses that model the subjective value (*SV*) of option *i* as a power law relationship, typically assuming that the target reward (*R*) is discounted relative to the target likelihood (*P*) by some power (*α*):

(1)
$$SV_{i}=P_{i}R_{i}{}^{\alpha }$$
Here, we assume that the reach bias observed is proportional to the ratio of the subjective values of the two targets:

(2)
$$Bias\propto \frac{SV_{1}}{SV_{2}}=\frac{P_{1}R_{1}{}^{\alpha }}{P_{2}R_{2}{}^{\alpha }}$$

$$=\left(\frac{P_{1}}{P_{2}}\right){\left(\frac{R_{1}}{R_{2}}\right)}^{\alpha }.$$
To be able to directly compare the biases for direct and intermediate reaches in this analysis, we scaled the bias on intermediate reaches (i.e., the initial reach direction) to be on the same range as direct reaches (between 0 and 1, since bias is measured as the proportion of reaches to the more rewarding target). Thus, initial reach direction on intermediate reaches was divided by the angular distance between the two targets (45°) and recentered ∼0.5.
Once the biases were rescaled, we then estimated *α* for both direct and intermediate reaches, for each iteration of our bootstrapped data. This was done by fitting a power law to the relationship between the reward ratio of the two targets and the bias divided by the likelihood ratio of the two targets as in Equation 2. Larger *α* values reflect less devaluation of target reward information relative to target likelihood information; or in other words, larger *α* values suggest that reward information exerts a stronger influence in determining the subjective value of the options, and hence more strongly influences the observed behavioral bias. Download Figure 5-1, EPS file.

10.1523/ENEURO.0503-21.2022.f5-2 Extended Data Figure 5-2 Reach-direction histograms for experiment 2. Initial reach direction for direct and intermediate reaches (data pooled across all participants), with varying reward and prospective-probability ratios. In all cases, the data have been aligned such that reaches to the more rewarded target are in the positive direction (rightward), and reaches to the more probable target are in the negative direction (leftward). As the relative reward ratio increases (left to right), reaches are biased toward the more rewarded target. Likewise, as the relative prospective-probability ratio increases (top to bottom), reaches are biased toward the more likely target. Fits reflect the average distribution parameters from bootstrapped fits to subsets of the pooled group distributions. Download Figure 5-2, EPS file.

10.1523/ENEURO.0503-21.2022.f5-3 Extended Data Figure 5-3 Estimation of biases in experiment 2. For direct reaches (***A***) and intermediate reaches (***B***), the effect of changing reward (top) or prospective-probability (left side) alone was fit by a linear regression. These regressions were then use to predict the reach direction bias observed when both reward and probability were changing (four small panels). Weighting the influence of probability and reward equally fit the data moderately well (blue squares); however, when the weights were allowed to vary, we obtained much better estimates (red circles). These weights indicated a much stronger influence of probability compared to reward for direct reaches, but the influence of probability and reward were weighted more similarly for intermediate reaches. Download Figure 5-3, EPS file.

### Prospective probability biases behavior analogously to frequency

One concern with modulating target frequency is that participants had to learn the relative frequencies between the two targets in order for that information to modulate behavior. Although the data suggest that varying the relative target frequencies did indeed change how people biased their responses, it is possible that participants did not notice the frequency manipulation and thus the biases observed were not actually representative of what happens when people consider the relative success likelihood of the options in their decisions. In addition, developing a frequency bias meant that for a given individual there was no way to counterbalance the more frequent target within a block; thus, there may have been idiosyncratic preferences, use-dependent repetition effects, or unintentional biomechanical biases unrelated to the frequency manipulation that influenced behavior. To address these concerns, we conducted a second experiment in which participants were explicitly shown the probability of the two targets alongside the reward values (Fig. 1*B*); each trial had no dependence on the previous one, and all reward and prospective-probability combinations were counterbalanced such that no target appeared more frequently within a block.

Despite these changes, on average participants exhibited analogous behavior to that of experiment 1 (see Extended Data Fig. 5-2). As before, bootstrapping was used to examine the relationship between direct and intermediate movement biases arising from varying target reward and success-likelihood ratios. For direct reaches (Extended Data Fig. 5-3*A*), we again observed a bias in the proportion of reaches aimed to the more rewarding target because of the reward ratio (slope = 0.009 ± 0.021 SD) and a bias in the proportion of reaches aimed to the more probable target because of the probability ratio (slope = −0.11 ± 0.010 SD). For intermediate reaches (Extended Data Fig. 5-3*B*), we also observed a bias in the initial reach direction because of the reward ratio (slope = 0.093 ± 0.23 SD) and a bias in the reach direction because of the probability ratio (slope = −0.45 ± 0.39 SD).

In attempting to predict the relative influence of probability and reward, we observed that as with experiment 1, direct reaches tended to be more strongly influenced by probability information compared with reward information [weight of probability, 0.76 ± 0.08 (SD); *t*_(769)_ = 85.27, *p *=* *0.00]. Intermediate reaches were also biased by probability more than reward [weight of probability, 0.55 ± 0.24 (SD), *t*_(769)_ = 5.68, *p *=* *1.93e-08], although as before the influence of probability was significantly greater for direct reaches compared with intermediate reaches (paired *t* test, *t*_(769)_ = 23.67, *p *=* *1.71e-93; Kolmogorov–Smirnov test, *p *=* *3.3902e-101; Fig. 5*B*). These results closely resemble those observed in experiment 1, replicating our prior findings that success likelihood and reward information are weighed to different extents in direct and intermediate reaches. This suggests that direct and intermediate reaches reflect qualitatively different aspects of behavior.

### Risk/reward-seeking attitude modulates behavioral biases

**Figure 6.**
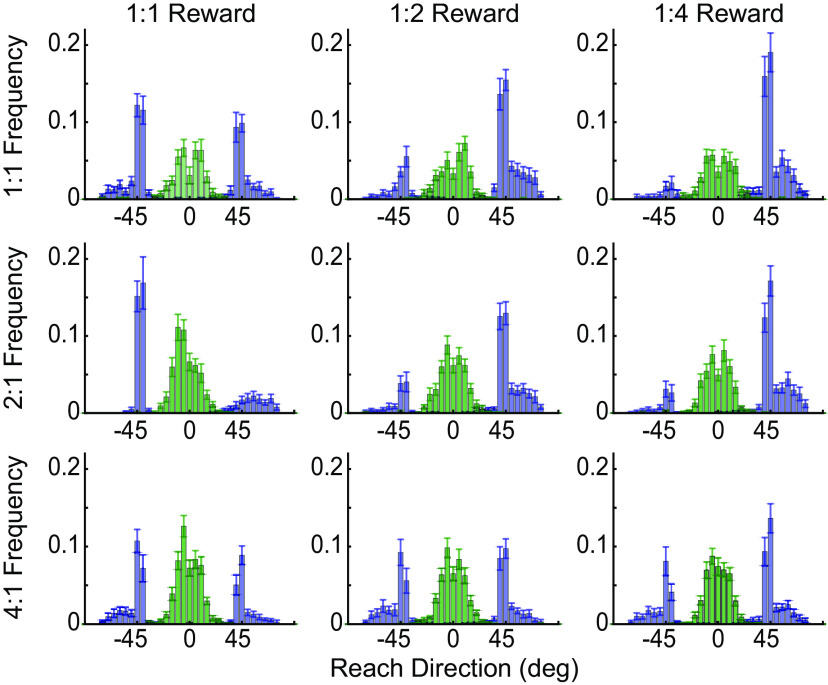
Average reach-direction histograms for experiment 3. For each condition, individual histograms were generated for each participant and then averaged. Bar height reflects the proportion of total reaches aimed in a particular direction (bins of 5°) for a given individual in that condition; error bars reflect SEM across participants. Data were labeled according to whether individuals intercepted the rectangular “intermediate” target to hit the far targets (green bars), or reached to one of the near “direct” targets (blue bars). As in Figure 3, data were sorted such that the positive reach directions were in the direction of the more rewarded target, while negative reach directions were in the direction of the more frequent target. As the relative reward ratio increased (left to right), reaches were biased toward the more rewarded target. Likewise, as the relative frequency ratio increased (top to bottom), reaches were biased toward the more likely target. The skew in the direct-reach distributions likely arose because the intermediate “box” option was quite wide and participants had to avoid hitting it when making a direct reach.

**Figure 7.**
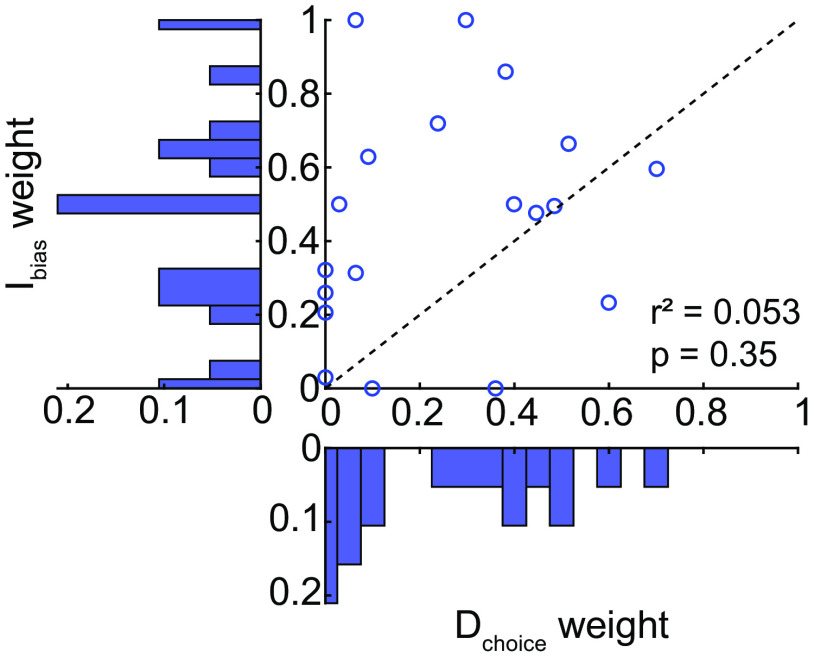
Comparison of individual likelihood-reward weight estimates for direct and intermediate reaches. As before, weights differed for direct and intermediate reaches, with no apparent systematic relationship evident. A parameter recovery analysis (Extended Data Fig. 7-1) suggests that this lack of correlation is likely not a result of the analysis method used to estimate these weights.

In experiments 1 and 2 (and historically in the literature), analyses were performed at the group level. This is largely because individual participants are idiosyncratic in their behavior: even in cases where the reward and success likelihood of the two targets are balanced, some participants produce only intermediate reaches, some produce only direct reaches, and some produce a mixture of both. Such variable responses make it challenging to directly compare behavior in direct and intermediate reaches within a single subject. Moreover, it is not clear what may be driving an individual’s particular choice of movement strategy. To investigate these questions, we designed a new experiment that encouraged individuals to generate a mixture of direct and intermediate reaches in every condition (Fig. 2*A*). Specifically, we gave participants an explicit “intermediate” movement option on each trial, and modulated the relative rewards on offer between the direct and intermediate choices to increase the chances of generating a particular type of response on a given trial (Fig. 2*A*; see Materials and Methods). This enabled us to estimate the relative weighting of frequency and reward on an individual-subject basis (Figs. 6, 7). Visual inspection revealed that individuals exhibited similar trends as observed in experiments 1 and 2: an increasing bias toward the more rewarded target as the reward ratio increased, and an increasing bias toward the more frequent target as the frequency ratio increased.

10.1523/ENEURO.0503-21.2022.f7-1 Extended Data Figure 7-1 Parameter recovery analysis to evaluate the weight-estimation method. In experiment 3, weights for direct and intermediate reaches were highly uncorrelated. To examine whether this finding arose because the weights were actually uncorrelated or because the method used to estimate the weights was unreliable, we performed a parameter recovery analysis. We observed good recovery of our simulation weights (compare the median recovered weight to the simulated weight, black open circles), for both (***A***) direct reaches, and (***B***) intermediate reaches. The dashed gray line reflects the line y = x; all median recovered weight values fall close to this line. ***C***, We also compared the recovered weights from direct and intermediate reaches to each other on each simulation repetition, where in each simulation the same weight value was used to generate both direct and intermediate reaches. Note that these points cluster also along the line y = x, suggesting that if we see a lack of relationship between the weights estimated on direct and intermediate reaches across participants, it is likely because these weights were indeed uncorrelated rather than because of an artifact introduced by the weight-estimation procedure.
For the parameter recovery analysis, we simulated the ability to accurately recover the underlying weights associated with frequency and reward biases. Biases were simulated in reach preference (direct reaches, *D_choice_*) and reach direction (intermediate reaches, *I_bias_*). These biases were determined by choosing a relative weighting of likelihood and reward. On each simulated trial, we calculated the preference for choosing the more frequent target based on the current trial’s frequency ratio and the reward ratio separately (e.g., if the frequency ratio is 2:1, the preference for choosing the more frequent target is 0.67). We calculated the distance this preference was from equal preference (i.e., 0.5), and scaled that distance by the predetermined frequency or reward weighting; this yielded a frequency-biased and reward-biased preference for each target:

$$Target_{1}\_preference_{frequency}=\left(\frac{frequency_{Target1}}{frequency_{Target1}+frequency_{Target2}}-0.5\right)*frequency\_weight+0.5$$

$$Target_{1}\_preference_{reward}=\left(\frac{reward_{Target1}}{reward_{Target1}+reward_{Target2}}-0.5\right)*reward\_weight+0.5.$$
Then, the objective utility (probability times reward) was computed for each of the two targets based on the scaled preference values:

$$EV\left[Target_{1}\right]=Target_{1}\_preference_{frequency}*Target_{1}\_preference_{reward}$$

$$EV\left[Target_{2}\right]=Target_{2}\_preference_{frequency}*Target_{2}\_preference_{reward}.$$
These expected values were normalized by the sum of the expected values for the two targets to yield the probability of choosing each target. On direct reaches, a random number was drawn; if the random number was lower than the probability of choosing Target 1, we recorded a direct-reach choice of Target 1; otherwise, we recorded a choice of Target 2. On intermediate reaches, we converted the probability of choosing Target 1 into a reach direction by multiplying by 30°, then subtracting 15° to center reaches about the midline. In this way, we could build a binary distribution of direct reaches and a continuous distribution of intermediate reaches, for every possible combination of frequency and reward ratios presented during experiment 3 (200 trials were simulated for each condition). Finally, we applied our weight estimation approach as described in Materials and Methods (computing regressions to estimate the relationship between frequency ratio and bias and reward ratio and bias separately, then finding the best weighting of these regressions to explain the bias observed when both frequency and reward ratios were not equal to 1) to recover the degree to which frequency was weighted across simulated trials (*D_choice_*_and_ *I_bias_*). We repeated this simulation for 100 iterations at each of nine frequency-reward weightings (0.1–0.9). We compared the recovered weighting to the actual weighting for both direct and intermediate reaches separately, as well as comparing the recovered weightings for direct reaches versus intermediate reaches (since on each repetition both direct and intermediate reaches were simulated from the same underlying weights). Download Figure 7-1, EPS file.

For each participant, we estimated the relative influence of the frequency and reward ratios on reach-direction biases, for both direct and intermediate reaches (Fig. 7). Across participants, frequency and reward influenced behavior to an unequal extent for direct reaches (weight of frequency, 0.25 ± 0.23 SD; *t*_(18)_ = −4.70, *p *<* *0.001), but not for intermediate reaches (weight of frequency, 0.46 ± 0.31 SD; *t*_(18)_ = −0.52, *p *=* *0.61). More importantly, the relative influence (weight) of frequency and reward for direct and intermediate reaches was significantly different from each other (paired *t* test, *t*_(18)_ = −2.72, *p* = 0.014). However, the relationship between these weights was not systematic across individuals (no correlation between the weights for direct and intermediate reaches, *r*^2^ = 0.053, *p* = 0.35). This lack of relationship between the weights estimated for direct and intermediate reaches was unlikely to be an artifact of the analysis method, as suggested by a parameter recovery analysis (see Extended Data Fig. 7-1).

**Figure 8.**
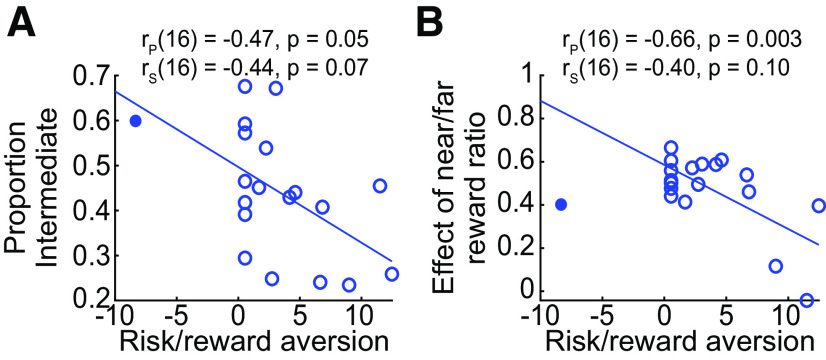
Risk/reward attitude correlated with individual differences in behavior. ***A***, Individuals who were more risk/reward averse (i.e., larger values on the abscissa) tended to generate a greater average proportion of intermediate movements, suggesting intermediate movements may reflect a strategy to maximize reward. Both Pearson (r_p_) and Spearman (r_s_) correlations are noted. The filled circle reflects the outlier participant, who was not included in the regression estimation. ***B***, Risk/reward-aversion tended to reduce the degree to which the relative reward ratio of near to far targets shifted the proportion of intermediate reaches produced (i.e., smaller ordinate values). The filled circle reflects the outlier participant, who was not included in the regression estimation.

Next, each individual’s risk/reward attitude was assessed to examine whether that could explain observed individual differences in behavior (Fig. 2*B*,*C*). We noted one outlier who exhibited unusually strong risk-seeking behavior; to ensure that this person did not skew our findings we removed this individual from further analysis. We then asked whether risk/reward attitude might relate to the proportion of direct or intermediate reaches generated (Fig. 8*A*). We observed that individuals who were more risk/reward-averse tended to generate fewer intermediate (i.e., more direct) movements on average across all conditions (Pearson *r*_(16)_ = −0.47, *p *=* *0.05; Spearman ρ_(16)_ = −0.44, *p *=* *0.07). Relatedly, we asked whether risk/reward attitude reflected an individual’s sensitivity to respond to the near-to-far reward manipulation. We noted that individuals who were more risk/reward-averse tended not to modulate their behavior as much in response to the near-to-far reward ratio (i.e., exhibited a smaller shift between making intermediate to direct reaches) as the near targets became relatively more rewarding compared with the far targets (Pearson *r*_(16)_ = −0.66, *p *=* *0.003; Spearman ρ_(16)_ = −0.40, *p *=* *0.10; Fig. 8*B*). Although these two effects were somewhat weak (i.e., nonsignificant Spearman correlations), together they suggested that risk/reward-averse individuals may be more likely to favor an intermediate-movement strategy.

Although the majority of our analyses were focused on the initial reach direction, the degree to which individuals failed to hit the correct target likely influenced behavior in this task. Individuals could miss targets for two reasons: they could choose the wrong target, or they could choose the correct target but still fail to earn the reward if they exhibited poor motor acuity. Previous work suggests that these two reasons for failure are dissociable in updating future choices (Green et al., 2010; Parvin et al., 2018; McDougle et al., 2019). To examine the effect of motor errors versus choice errors in our task, we asked whether these factors and risk/reward attitude related to the average behavior during this task.

We found that when individuals made a greater fraction of choice errors during intermediate compared with direct reaches, they tended to favor making direct reaches (β = −1.29 ± 0.57, *t*_(12)_ = −2.24, *p *=* *0.044); moreover, there was a significant interaction between choice errors and risk/reward attitude (β = 0.14 ± 0.06, *t*_(12)_ = 2.40, *p *= 0.033), suggesting that incorrect choices have a smaller effect on behavior as people become more risk/reward averse. Although motor errors also tended to have similar effects on behavior, neither the main effect (β = −1.71 ± 0.85, *t*_(12)_ = −2.02, *p *=* *0.07) nor the interaction with risk/reward attitude (β = 0.16 ± 0.16, *t*_(12)_ = 0.99, *p *=* *0.34) were significant.

We also asked whether motor and choice errors were related to the relative weighting of success likelihood and reward for direct and intermediate reaches separately. We observed a significant relationship between choice errors and the success-likelihood/reward weights for direct reaches (β = 2.11 ± 0.84, *t*_(15)_ = 2.52, *p *=* *0.02) but not for intermediate reaches (β = −0.34 ± 1.39, *t*_(15)_ = −0.25, *p *=* *0.81). Again, motor errors had no effect on either set of weights (*p* > 0.12), nor did the two types of errors interact (*p* > 0.14). This makes sense since direct reaches involved committing to a target before movement onset with no opportunity to change that decision mid-flight, and thus were more likely to depend on choice-success information (e.g., success likelihood and choice errors). These findings thus suggest that the initial reach direction in this task is primarily driven by choice errors rather than motor errors, and that errors largely affect behavior on direct but not intermediate reaches.

Together, the findings from experiment 3, taken alongside the results from experiments 1 and 2 showing that intermediate movements were more strongly biased by reward compared with direct movements, suggest that intermediate and direct movements may represent attempts to maximize reward versus success, respectively, and this behavior may reflect the degree to which an individual is risk/reward-seeking.

## Discussion

This study examined two key questions regarding how people respond when faced with multiple potential options. The first question asked how individuals weigh reward and success-likelihood information (i.e., exogenous and endogenous rewards) in determining the relative desirability of each option, and whether any preference biases that arose would be analogous for both direct and intermediate reaches. We showed that the decision biases induced by varying the desirability of the two targets were not analogous for direct and intermediate reaches. Specifically, at least for experiments 1 and 2, direct reaches were primarily influenced by target likelihood while intermediate reaches were more influenced by reward. Moreover, the degree to which individuals responded to choice errors in this task also differed between direct and intermediate reaches. This importantly suggests that intermediate reaches do not arise from the averaging of direct-reach plans. Instead, these findings are consistent with and extend previous work demonstrating that intermediate movements are deliberately adopted as a distinct motor strategy to improve task performance (Wong and Haith, 2017; Dekleva et al., 2018; Alhussein and Smith, 2021), in this case, to maximize rewards. More broadly, these findings reveal that different behaviors, even when generated within the context of the same task, can be differentially motivated by reward or success; or equivalently, that rewards (money) and success (being correct) can influence behavior differently. This highlights the fact that not only are reward and success likelihood generally subjectively viewed as differentially important in determining a movement response (see Extended Data Fig. 5-1; Kahneman and Tversky, 1979; Onagawa et al., 2019), but moreover that their subjective importance changes depending on the strategy with which one chooses to approach a given task.

Direct and intermediate movement strategies therefore appear to be optimizing different things: task success versus reward outcomes, respectively. Such an optimization makes sense: on direct reaches, individuals must commit to a decision before movement onset; hence the best approach is to choose the target that is more likely to be correct, even at the cost of occasionally missing large, rare rewards. Direct reaches reflect a relatively low-effort, low-risk strategy to successfully hit the correct target most of the time, and rely in large part on keeping track of average target frequencies and choice errors. On the other hand, intermediate reaches allow individuals to delay commitment to a particular option until more information can be acquired, and affords the ability to change one’s mind. Because this strategy reduces the risk of failure, individuals can instead focus on maximizing rewards; hence it is sensible to consider both the success likelihoods and the rewards of the two options. However, intermediate movements are also more effortful to complete: they necessarily require constant monitoring of the target options throughout the movement to identify the correct target as soon as it is revealed, and they always require a motor correction to be generated midway through the movement. This taxes both cognitive and physical resources. It has previously been shown that the prospect of larger rewards can offset the cost of physical and mental effort (Dixon and Christoff, 2012; Schmidt et al., 2012; Manohar et al., 2015; Summerside et al., 2018). Interestingly, our findings suggest that reward may have a stronger influence than success in offsetting the cost of moving, hence reward prospect seems to largely drive an intermediate-movement strategy. Thus, direct and intermediate reaches appear to reflect distinct movement strategies to respond to decision uncertainty, residing on opposite ends of the effort-reward (and risk-reward) trade-off.

We note that the groupwise results of experiment 3 differ from this interpretation, although this could be attributed to two key differences of this Experiment from the other two. First, participants in experiment 3 may have been less motivated by the rewards on offer, given that rewards in experiments 1 and 2 were generally larger because of the tendency for individuals to be risk/reward averse. In experiment 3, large rewards were primarily presented when the near-to-far reward ratio favored direct reaches, which could cause intermediate reaches to appear less reward-sensitive. Second, participants were made explicitly aware of the choice between direct and intermediate reaches in experiment 3, which may have influenced people’s strategies. Thus, while the design of experiment 3 allowed us to avoid having to classify reaches as direct or intermediate in a *post hoc* fashion and to examine the relationship between motor strategy and risk/reward attitude, it may also have impaired the interpretability of our group results. Nevertheless, what is consistent across all three Experiments is that the relative weighting of success likelihood and reward differ for direct and intermediate reaches.

Our second question examined whether we could explain why individuals preferentially generate direct and intermediate movements to different extents when asked to reach under the same conditions of target uncertainty. Given that direct and intermediate reaches seem to be distinct strategies favoring success or reward, respectively, we hypothesized that individual risk/reward attitudes might influence how individuals choose to respond (Wong and Haith, 2017). At first glance, one might think that because intermediate movements enable individuals to change their mind, this might be the strategy preferred by more risk-averse individuals. Instead, we found evidence suggestive of the opposite result: the more risk/reward-seeking the individual, the more they tended to favor making intermediate reaches. Since intermediate movements were more strongly influenced by reward relative to direct reaches, however, this makes sense: reward-seeking individuals preferentially adopt a strategy that allows them to maximize their potential rewards.

Note that although intermediate movements have the potential to also improve success at hitting the correct target, not every intermediate movements is successful (Wong and Haith, 2017); thus, intermediate movements are not necessarily the more risk-averse movement strategy. Additionally, as noted above, they are also the more effortful strategy, and thus by subjectively valuing rewards to a greater extent individuals may be more likely to perceive that they are achieving an adequate offset for this additional effort cost (Klein-Flügge et al., 2016; Galaro et al., 2019). Consistent with these ideas, risk/reward-seeking individuals also responded more strongly to the reward manipulation in experiment 3 that was designed to modulate the production of intermediate versus direct reaches (i.e., the relative rewards offered for near vs far targets). In contrast, individuals who were more risk-averse and less reward-seeking seemed to favor making direct reaches, suggestive of an effort to maximize success at hitting the correct target. Although it is possible that these observed relationships were affected by outliers since the Spearman correlations did not reach significance, we do note the consistency of the relationship trend between risk/reward attitude and both the proportion of intermediate movements generated as well as the degree of responsivity to the near-to-far reward ratio (which modulates the relative number of intermediate movements generated). Thus, these findings, while not definitive, are nonetheless consistent with the idea that an intermediate movement strategy is more likely to be adopted by someone who is more risk/reward seeking.

A key observation in our findings is that success likelihood and reward are weighted differently for direct or intermediate reaches. There are two possible explanations for this. On one hand, success likelihood and reward information could first dictate the movement strategy (direct or intermediate), but then success likelihood and reward would need to be re-weighted differently when choosing a target. Alternatively, the choice of movement strategy could surprisingly have occurred first, and in turn influenced how success likelihood and reward information were employed to determine target choice. While either explanation is possible, the latter seems more parsimonious. Thus, our results imply a hierarchical decision process in which, in response to choice uncertainty, individuals first select a movement strategy (i.e., whether to generate a direct or intermediate reach). This movement strategy in turn may then modulate the way in which reward and success-likelihood information bias reach choice. Such a hierarchical decision structure has previously been argued to be favorable for action selection (Rosenblatt and Payton, 1989; Tyrrell, 1993). Interestingly, such a hierarchy results in two consequences that are relevant here. First, it allows relevant sensory information to be introduced at lower levels of the hierarchy after some decisions have already been made, avoiding a sensory bottleneck and maintaining a greater amount of information in the network. Second, it enables individuals to identify and select “compromise candidates,” or options that are favorable given overriding constraints. In our case, the compromise candidate is reflected in the intrinsic movement bias arising after a decision strategy has been adopted; moreover, subject-specific movement-strategy preferences can be determined according to an individual’s risk/reward-seeking propensity without first needing to consider the reward and success-likelihood values on offer in a given trial. Indeed, we note that while success-likelihood and reward information seem most relevant for setting movement biases once a choice of direct or intermediate has been made, risk/reward attitude modulates decisions at multiple levels of the hierarchy. It affects the preference for making direct versus intermediate reaches, and subsequently influences the relative weighting of success likelihood and reward information in determining which option is more desirable. Importantly, such a hierarchical structure suggests that the subjective value placed on action choices—in neuroeconomics terms, their utility—not only influences movement decisions about where to reach (Wolpert and Landy, 2012), but is itself influenced in turn by movement decisions about how to reach. Although surprising, this is consistent with recent work showing that selecting motor parameters like movement speed can influence more cognitive processes (i.e., the speed of decision-making; Carsten et al., 2022).

While some amount of effort has been made to investigate the neural underpinnings of direct and intermediate movements (Cisek and Kalaska, 2002; Cisek, 2007; Dekleva et al., 2018), much of this work thus far has focused on premotor cortex. In contrast, efforts to study the representation of subjective value and relative choice desirability have largely investigated midbrain structures including the basal ganglia and ventral tegmenal area, and associated areas of frontal cortex (Samejima et al., 2005; Trepel et al., 2005; Croxson et al., 2009; Fox and Poldrack, 2009; Levy et al., 2010; Schmidt et al., 2012; Klein-Flügge et al., 2016; Doi et al., 2020; Shin et al., 2021). Intriguingly, however, there is some suggestion that parietal reach region in the posterior parietal cortex might be a potential area where information about motor goals and goal desirability converge (Trepel et al., 2005). Specifically, neurons in parietal reach region have been shown to modulate according to both the rewards and success likelihoods of action choices as if computing target utility (Platt and Glimcher, 1999); additionally, this region seems capable of simultaneously representing multiple potential movement goals (Klaes et al., 2011). Subjective utility has also been shown to modulate cortical excitability in M1 and directly influence behavior (Galaro et al., 2019). This suggests multiple points at which information about target desirability can interact with decisions about where to reach, in line with the notion of a hierarchical decision structure (Rosenblatt and Payton, 1989; Tyrrell, 1993).

In conclusion, our data suggest that when moving in the face of uncertainty, individuals deliberately adopt a motor strategy in line with their proclivity to be risk/reward-seeking. In particular, this proclivity influences whether individuals prefer to commit to a guess before moving (i.e., generating direct movements), versus hedging one’s bets and delaying commitment until later in the movement (i.e., generating intermediate movements). Our findings support the idea that intermediate movements are planned as part of a deliberate movement strategy to maximize reward, distinct from that underlying the planning of direct reaches which seeks to maximize success. Moreover, the choice of movement strategy appears to influence the manner in which individuals evaluate the subjective value of the available options. Together, this suggests that in the face of uncertainty, people trade off maximizing task success and earning monetary rewards depending on their individual risk/reward attitudes, and these decisions influence both the choice of movement strategy as well as the apparent subjective value of the available options.

## Synthesis

Reviewing Editor: Ifat Levy, Yale University School of Medicine Decisions are customarily a result of the Reviewing Editor and the peer reviewers coming together and discussing their recommendations until a consensus is reached. When revisions are invited, a fact-based synthesis statement explaining their decision and outlining what is needed to prepare a revision will be listed below. The following reviewer(s) agreed to reveal their identity: Christian Seegelke.

In this paper, the authors dig deeper into the well-known finding that people often hedge their bets when making rapid reaching decisions between multiple potential movement targets (i.e., reaching between them and delaying a final decision). In that literature, it is also known that this “late-swerving” strategy is sometimes replaced by a simpler all-or-none strategy, where a single target is decided upon before movement initiation (i.e., reaching directly to one of two potential targets). While much previous work has shed light on how intermediate movements reflect decision optimization, the trade-off between the two distinct strategies has not been investigated as deeply. Indeed, there is apparently a lot of variability between individuals in how they calibrate their use of these two movement strategies. Here, over 3 experiments, the authors suggest that these two strategies are actually qualitatively distinct approaches to motor planning under uncertainty, rather than two sides of a single “reward optimization” coin. The authors first show that the two strategies are differentially sensitive to reward likelihood versus reward magnitude. Second, they perform a novel experiment that pits the strategies against one another, allowing for an individual difference analysis; this analysis suggests that the trade-off between these two distinct strategies is related to intrinsic risk aversion (i.e., at the level of simple betting task behavior).

Overall, this is a very strong well-written paper. The gaps in the literature that it fills are clear, the methods and analyses are sound and rigorous (and clearly described), and the conclusions are justified by the data. The paper provides novel evidence to the debate as to whether intermediate reaches reflect (low-level) motor averaging or a deliberate strategy to improve task performance, clearly favoring the latter hypothesis. Reviewers had, however, several comments that need to be addressed, as detailed below.

Major point:

-One of the main conclusions of the manuscript is that intermediate movements reflect a strategy to maximize reward, whereas direct reaches aim at maximizing task success. It seems, however, that not all data are compatible with such an interpretation. Specifically, whereas the frequency/probability manipulation clearly affected direct reaches in Experiment 1 and 2 (see Figure 3 left panel, extended Figure 5-2; weights of .66 and .76, respectively), judging from visual inspection of Figure 6, increasing the frequency ratio had little effect on either strategy. In addition, in Experiment 3, the weights seem to be reversed compared to Experiments 1 and 2, i.e., for direct reaches the weight for frequency was .32 (significantly lower than .5) and .62 for intermediate reaches. This indicates that in Experiment 3, direct reaches were influenced more by reward, whereas intermediate reaches were more influenced by frequency. These findings stand in stark contrast to the results of Experiment 1 and 2 and to the conclusion drawn by the authors and need to be explained and further discussed.

-What type of correlation was used? The correlational analyses seem to be compatible with the overall conclusion, but if it was Pearson correlation, then it is highly sensitive to outliers (e.g., Rousselet & Pernet, 2012; Aggarwal & Ranganathan, 2016) and estimates of correlation analyses are often inaccurate in small samples (Schönbrodt & Perugini, 2013). Specifically, as shown in Figure 8B, many data point are clustered in the “lower left part” of the figure, and removing the “top” data point may have a massive influence.

-The difference between two forms of “success” - one at the level of getting a reward or not getting anything, and the other at the level of motor accuracy (reaching a desired location in an ecological sense) is ignored. However, this seems like an apropos topic that is worthy of at least mentioning (e.g. see Green et al., 2010; Parvin et al., 2018; McDougle et al., 2019), if not directly assessing in some additional analyses. For instance, are there differences in the final endpoint errors that may say something about the tradeoff between the two strategies (e.g., a cost of greater error in intermediate reaches that is “made up for” by reward maximization)? Or perhaps the percentage of out-of-bounds/timed-out trials during intermediate versus direct trials (indeed, there seems to be a decent proportion of these)? Or, relating to Trommershäuser and Landy’s excellent work, a relationship between subjects’ intrinsic motor noise and the intermediate/direct tradeoff? It seems that it may be the case that motor error is factored into the high-level strategy decision, thus linking the two strategies at the top level of the hierarchy (rather than them being totally unrelated).

-Could the authors elaborate a bit on why they think that participants first select a movement strategy and this strategy then influences the computation of relative weighing of reward and likelihood. Couldn’t it theoretically be also the other way around, i.e., the reward and likelihood influence the selected strategy?

Minor points:

-The computation of (Tcommit) was a bit confusing, e.g. “as the time when the reach direction was equal to the instantaneous target direction.” This can’t be exact, right? What are the bounds of this analysis? Was binning used? A fixed interval?

- Also, reach direction is calculated as velocity vector but a position vector is shown in Figure 1C. Please revise or highlight in the figure caption to explain why TChoose is not at the point in time where the green line crosses the blue line

- P.13 : The time point where the decision is made is called TCommit in the text but TChoose in Figure 1C

-Figure 4 is rather cluttered and hard to parse

-"risk-adverse” is written sometimes but should be “risk-averse.”

-Figure 6 top right should probably read 1:4 instead of 4:1.

-Rhos and p-values could be more prominent, i.e. printed on the correlation figures themselves (e.g., Figs 7 & 8).

- Different terms are used for similar concepts, e.g., success likelihood/ endogenous reward/ endogenous success; reward/ attainable reward/ exogenous reward; likelihood/probability. Readers may wonder whether these different terms mean the same or (slightly) different things. Please consider using consistent terminology.

- P. 1 l. 6-9: Two alternatives are entertained (i.e., distinct movement strategies vs. general response) but outcome is only proposed for one - consider rephrasing.

- P. 2 l. 30: The word “determined” may suggest some kind of causality, whereas this statement relates to a correlational analysis

- P- 5. l. 100: The authors state that in Experiment 3, 18 of 27 participants generated direct and intermediate reaches in each condition. What about participants’ behavior in Experiment 1 and Experiment 2?

- P. 9 l. 161-169: More information on the Utility Test would be helpful. The test is clear in Experiment 3 (visualized in Figure 2C) but it is not clear if it was the same (or similar) in Experiments 1 and 2.

- P. 12 l. 227-229: If the Utility Test was not used to set reward values, what were the exact values used in Experiment 3?

- P. 12 l. 236: What was the rationale for using different velocity threshold values in Exp1/2 and Exp3?

- P. 13 l. 238-245: Why were different exclusion criteria used for the three experiments?

- P. 17 l. 330: Figure “5C” should be “2C"

- P. 18 l. 355: For consistency, consider sticking to “more” instead of “less” and “fewer"

- P. 18 l. 356-357: The sentence in brackets may be distracting

- P. 23 l. 438-440: The increasing bias to more frequent targets is hard to spot in Figure 6

- P. 25: You present positive correlation between risk-seeking and proportion of intermediate movements in the text but show negative correlation in Figure 8A between risk-aversion and proportion of intermediate movements. Consider using consistent terms in the text and figure.

- Extended Fig 5-2: “1:5” instead of “5:1” reward; same for Fig6. “1:4” instead of “4:1"

- Fig 8: Please indicate that lower values denote risk-seeking and higher values risk-averse behavior

- It would also be helpful to note the outlier in Fig 8

## Author Response

Dear Dr. Levy,

Thank you to the reviewers for their thoughtful comments. We hope that we have addressed all

of their concerns in a satisfactory manner, as described below, and think that these edits have much

improved our manuscript.

Sincerely,

The Authors

Synthesis Statement for Author (Required):

In this paper, the authors dig deeper into the well-known finding that people often hedge their bets

when making rapid reaching decisions between multiple potential movement targets (i.e., reaching

between them and delaying a final decision). In that literature, it is also known that this “late-swerving”

strategy is sometimes replaced by a simpler all-or-none strategy, where a single target is decided upon

before movement initiation (i.e., reaching directly to one of two potential targets). While much previous

work has shed light on how intermediate movements reflect decision optimization, the trade-off

between the two distinct strategies has not been investigated as deeply. Indeed, there is apparently a

lot of variability between individuals in how they calibrate their use of these two movement strategies.

Here, over 3 experiments, the authors suggest that these two strategies are actually qualitatively

distinct approaches to motor planning under uncertainty, rather than two sides of a single “reward

optimization” coin. The authors first show that the two strategies are differentially sensitive to reward

likelihood versus reward magnitude. Second, they perform a novel experiment that pits the strategies

against one another, allowing for an individual difference analysis; this analysis suggests that the trade-off between these two distinct strategies is related to intrinsic risk aversion (i.e., at the level of simple

betting task behavior).

Overall, this is a very strong well-written paper. The gaps in the literature that it fills are clear, the

methods and analyses are sound and rigorous (and clearly described), and the conclusions are justified

by the data. The paper provides novel evidence to the debate as to whether intermediate reaches

reflect (low-level) motor averaging or a deliberate strategy to improve task performance, clearly

favoring the latter hypothesis. Reviewers had, however, several comments that need to be addressed,

as detailed below.

We appreciate the reviewers’ positive comments about our manuscript.

Major point:

- One of the main conclusions of the manuscript is that intermediate movements reflect a strategy to

maximize reward, whereas direct reaches aim at maximizing task success. It seems, however, that not all

data are compatible with such an interpretation. Specifically, whereas the frequency/probability

manipulation clearly affected direct reaches in Experiment 1 and 2 (see Figure 3 left panel, extended

Figure 5-2; weights of .66 and .76, respectively), judging from visual inspection of Figure 6, increasing

the frequency ratio had little effect on either strategy. In addition, in Experiment 3, the weights seem to

be reversed compared to Experiments 1 and 2, i.e., for direct reaches the weight for frequency was .32

(significantly lower than .5) and .62 for intermediate reaches. This indicates that in Experiment 3, direct

reaches were influenced more by reward, whereas intermediate reaches were more influenced by

frequency. These findings stand in stark contrast to the results of Experiment 1 and 2 and to the

2

conclusion drawn by the authors and need to be explained and further discussed.

We agree that the difference between Experiment 3 and Experiments 1&2 is puzzling. We carefully went

back and checked over our analysis, and caught a mistake in Experiment 3 where not all the data were

being aligned properly such that in some cases the direction of the more frequent/rewarding targets

were reversed. We therefore carefully re-analyzed our data from Experiment 3 to address this issue.

However, in general our results did not qualitatively change. We also checked if this was an artifact of

using a population-level analysis for Experiments 1&2, but this could not account for the difference in

Experiment 3 either. Thus we are fairly confident that behavior in Experiment 3 does differ from that of

Experiments 1&2. We note two potential explanations for this. First, in Experiment 3 reward values were

not adjusted in a subject-specific manner based on each person’s utility (subjective value of reward).

While this allowed us to ask how utility related to choice behavior, it also meant that each individual was

differently responsive to the offered rewards. Since individuals across all experiments tended to be risk-averse (and hence adjusted reward offers were higher in Experiments 1&2), participants in Experiment 3

may therefore have been less motivated by the offered rewards unless rewards were large (e.g., when

the near-to-far reward ratio favored making direct reaches). This could have caused a greater apparent

weighing of likelihood information on intermediate reaches compared to Experiments 1&2.

The second key difference between Experiments 1&2 and Experiment 3 is that the choice between

direct and intermediate reaches was made explicit to participants. In Experiments 1&2, participants may

not have been as cognizant of the pros/cons of making this choice. This is certainly a limitation of the

design of Experiment 3, although it enabled us to avoid the problem of post hoc classification of reaches

as direct or intermediate.

Regardless, across all three experiments we see that regardless of what the specific weighting of

likelihood and reward within a given experiment, these weights are different for direct and intermediate

reaches. We now discuss these limitations of our work in the discussion:

"We note that the results of Experiment 3 differ from this interpretation, although this could be

attributed to two key differences of this Experiment from the other two. First, participants in

Experiment 3 may have been less motivated by the rewards on offer, given that rewards in Experiments

1 and 2 generally were increased in response to the tendency for individuals to be risk/reward averse. In

Experiment 3, large rewards were primarily presented when the near-to-far reward ratio favored direct

reaches, would could cause intermediate reaches to appear less reward-sensitive. Second, participants

were made explicitly aware of the choice between direct and intermediate reaches in Experiment 3,

which may have changed people’s strategies. Thus, while the design of Experiment 3 allowed us to avoid

having to classify reaches as direct or intermediate in a posthoc fashion and to examine the relationship

between motor strategy and risk/reward attitude, it may also have limited our interpretability of our

results. Nevertheless, what is consistent is that across all three Experiments, the relative weighting of

likelihood and reward differ for direct and intermediate reaches.”

- What type of correlation was used? The correlational analyses seem to be compatible with the overall

conclusion, but if it was Pearson correlation, then it is highly sensitive to outliers (e.g., Rousselet &

Pernet, 2012; Aggarwal & Ranganathan, 2016) and estimates of correlation analyses are often

inaccurate in small samples (Schönbrodt & Perugini, 2013). Specifically, as shown in Figure 8B, many

data point are clustered in the “lower left part” of the figure, and removing the “top” data point may

3

have a massive influence.

We had originally computed the coefficient of determination (R2

), which is equivalent to the Pearson

correlation. We agree, however, that this method may be overly sensitive to outliers. To address this

concern, we now report both the Pearson and Spearman correlations. Following our re-analysis (see

above), we found that while the two correlations with risk-seeking tendency were significant according

to Pearson correlations, they failed to reach significance for Spearman correlations. However, we note

that the direction of these two correlations are consistent with each other, which adds some added

support. We have updated our results, and have revised our conclusions accordingly:

"Although it is possible that these observed relationships were affected by outliers since the Spearman

correlations did not reach significance, we do note the consistency of the relationship trend between

risk/reward attitude and both the proportion of intermediate movements generated as well as the

degree of responsivity to the near-to-far reward ratio (which modulates the relative number of

intermediate movements generated). Thus these findings, while not definitive, are nonetheless

consistent with the idea that an intermediate movement strategy is more likely to be adopted by

someone who is more risk/reward seeking.”

- The difference between two forms of “success” - one at the level of getting a reward or not getting

anything, and the other at the level of motor accuracy (reaching a desired location in an ecological

sense) is ignored. However, this seems like an apropos topic that is worthy of at least mentioning (e.g.

see Green et al., 2010; Parvin et al., 2018; McDougle et al., 2019), if not directly assessing in some

additional analyses. For instance, are there differences in the final endpoint errors that may say

something about the tradeoff between the two strategies (e.g., a cost of greater error in intermediate

reaches that is “made up for” by reward maximization)? Or perhaps the percentage of out-of-bounds/timed-out trials during intermediate versus direct trials (indeed, there seems to be a decent

proportion of these)? Or, relating to Trommershäuser and Landy’s excellent work, a relationship

between subjects’ intrinsic motor noise and the intermediate/direct tradeoff? It seems that it may be

the case that motor error is factored into the high-level strategy decision, thus linking the two strategies

at the top level of the hierarchy (rather than them being totally unrelated).

We thank the reviewers for this very helpful suggestion. We agree that there are two different forms of

"success” - choosing the correct target versus hitting the chosen target with proper acuity. It is certainly

possible that these two types of success affect behavior in our task in different ways. Since our task

design makes it challenging to conduct a trial-by-trial analysis, we instead examined average behavior.

Specifically, we quantified the difference in the proportion of choice and motor errors for direct and

intermediate reaches for each individual in Experiment 3, and asked whether this difference related to

three aspects of behavior: (1) the average proportion of intermediate reaches produced, (2) the

likelihood/reward weight for direct reaches, and (3) the likelihood/reward weight for intermediate

reaches. We found that in general, choice errors but not motor errors modulated behavior. People who

exhibited more choice errors on intermediate reaches tended to generate more direct reaches overall.

In addition, the likelihood/reward weight was modulated by choice errors for direct but not

intermediate reaches, and neither weighting was affected by motor errors. Together, these findings

suggest that choice errors are primarily the error of interest in this paradigm, and that they

predominantly affect behavior on direct reaches when people must commit to a target choice before

moving. We now include these findings in the results and discussion sections.

4

- Could the authors elaborate a bit on why they think that participants first select a movement strategy

and this strategy then influences the computation of relative weighing of reward and likelihood.

Couldn’t it theoretically be also the other way around, i.e., the reward and likelihood influence the

selected strategy?

We apologize for the lack of clarity, and now attempt to provide a clearer explanation of our reasoning

in the discussion. The key observation is that that the degree to which reward and likelihood bias reach

direction differs depending on whether individuals make direct or intermediate reaches. If, as the

reviewers suggest, reward and likelihood instead dictated motor strategy, reward and likelihood would

need to be weighted twice (or need to exist with two distinct set of weightings): once to determine the

strategy choice, and then again in order for the bias in reach direction to be different on direct and

intermediate reaches. Instead, if the strategy were selected first, reward and likelihood information

would only need to be weighted once per trial (conditional on movement strategy). While either

explanation is possible, we suggest that the latter seems more parsimonious. We also note the similarity

of the directionality of this effect (i.e., motor planning influencing cognitive decisions) to a recent

preprint suggesting that planning movements of different speeds affects the speed of decision-making

(Carsten et al. bioRxiv 2022).

"A key observation in our findings is that success likelihood and reward are weighted differently for

direct or intermediate reaches. There are two possible explanations for this. On one hand, success

likelihood and reward information could first dictate the movement strategy (direct or intermediate),

but then success likelihood and reward would need to be re-weighted differently when choosing a

target. Alternatively, the choice of movement strategy could surprisingly have occurred first, and in turn

influenced how success likelihood and reward information were employed to determine target choice.

While either explanation is possible, the latter seems more parsimonious.”

Minor points:

-The computation of (Tcommit) was a bit confusing, e.g. “as the time when the reach direction was

equal to the instantaneous target direction.” This can’t be exact, right? What are the bounds of this

analysis? Was binning used? A fixed interval?

- Also, reach direction is calculated as velocity vector but a position vector is shown in Figure 1C. Please

revise or highlight in the figure caption to explain why TChoose is not at the point in time where the

green line crosses the blue line

- P.13 : The time point where the decision is made is called TCommit in the text but TChoose in Figure 1C

We apologize for the confusion regarding the method used to classify reaches as direct or intermediate.

We also apologize for the mislabeling, and will consistently use Tcommit in both the text and Figure.

Since the position of the hand changes over time, the direction of the two targets relative to the current

hand position also changes as the reach progresses. We compute for each sample what the direction of

the hand is, and the range of angles spanned by the widths of each of the two targets relative to the

current hand position. Since our data are sampled at a rate of 1000 Hz, we can estimate with fairly high

temporal resolution the first time (in ms) when the hand was directed toward the target, from the time

of movement onset to the time the movement ended. We do not use any binning to perform this

analysis.

5

We also apologize for the confusion regarding the calculation of reach direction. The instantaneous

reach direction is the direction of the vector tangent to the hand position trace at each point in time,

and the tangent vector of reach direction is the velocity vector. We have now tried to clarify this in the

Methods. Thus in Fig 1C, we plot the angle of the velocity vector, which is equivalent to the

instantaneous reach direction (we are not plotting a position vector). Also in Fig. 1C, the blue line

represents the center of the target, while the shaded blue region represents the angular width of the

target from the perspective of the current hand location. TCommit is the time when the hand is first

directed within the bounds of the target, or when the green line crosses into the blue shaded region. We

now clarify this in the figure legend.

-Figure 4 is rather cluttered and hard to parse

We apologize for the difficulty in parsing Figure 4. We have struggled to find a satisfactory way to

illustrate this in a concise and clear manner. We have now adjusted the symbols in the figure and added

additional text/lines for clarity, and hope that it is now more readable.

-Rhos and p-values could be more prominent, i.e. printed on the correlation figures themselves (e.g.,

Figs 7 & 8).

We have updated these figures to reflect our updated analyses, and now report Pearson and Spearman

rho and p values on the figures per the reviewers’ suggestion.

- Different terms are used for similar concepts, e.g., success likelihood/ endogenous reward/

endogenous success; reward/ attainable reward/ exogenous reward; likelihood/probability. Readers

may wonder whether these different terms mean the same or (slightly) different things. Please consider

using consistent terminology.

We apologize for the inconsistency. We now use “success likelihood” and “reward” consistently

throughout, and provide operational definitions of these terms in the introduction.

- P. 1 l. 6-9: Two alternatives are entertained (i.e., distinct movement strategies vs. general response)

but outcome is only proposed for one - consider rephrasing.

We agree this was not well-written, and have rephrased this sentence.

- P. 2 l. 30: The word “determined” may suggest some kind of causality, whereas this statement relates

to a correlational analysis

We have changed this word to better reflect the correlational nature of our analysis.

- P- 5. l. 100: The authors state that in Experiment 3, 18 of 27 participants generated direct and

6

intermediate reaches in each condition. What about participants’ behavior in Experiment 1 and

Experiment 2?

We now report the number of individuals who reliably generated direct and intermediate reaches in

Experiments 1 and 2:

"In Experiment 1 only 13 out of 24 participants reliably made both direct and intermediate reaches (i.e.,

at least 5% of each type of reach) in every block, and in Experiment 2 only 8 out of 17 participants

reliably made both direct and intermediate reaches. Hence, data from these experiments were analyzed

at the population level.”

- P. 9 l. 161-169: More information on the Utility Test would be helpful. The test is clear in Experiment 3

(visualized in Figure 2C) but it is not clear if it was the same (or similar) in Experiments 1 and 2.

We apologize for the lack of clarity. The Utility Test was indeed conducted in the same manner across all

three experiments; we now note this explicitly in the Methods. The only difference is that for

Experiments 1 and 2 the reward values were adjusted prior to the session according to the outcome of

the Utility Test.

- P. 12 l. 227-229: If the Utility Test was not used to set reward values, what were the exact values used

in Experiment 3?

We now clarify this in the methods; in Experiment 3, we used the same reward values for all participants

with a base value of 5 cents.

- P. 12 l. 236: What was the rationale for using different velocity threshold values in Exp1/2 and Exp3?

Different velocity thresholds were applied because we were less concerned with needing to robustly

classify movements as direct or intermediate in a posthoc manner; thus we could be more liberal with

regard to when we considered movement onset to occur by using a timepoint that was earlier in the

movement. We now note this in the methods.

- P. 13 l. 238-245: Why were different exclusion criteria used for the three experiments?

We now note in the Methods why the exclusion criteria differed. Because Experiment 2 required people

to observe the stimuli to identify the relative probabilities on each trial, in the Experiment we placed the

time limit on people’s movement time rather than on the total time from target appearance; hence the

exclusion criteria in Experiment 2 was movement-time based. The changes for Experiment 3 reflected

the fact that people sometimes exaggerated their reach direction to avoid the central rectangle when

making direct reaches, and to account for the longer distance/duration of intermediate reaches .

- P. 17 l. 330: Figure “5C” should be “2C"

-"risk-adverse” is written sometimes but should be “risk-averse.”

7

-Figure 6 top right should probably read 1:4 instead of 4:1.

- Extended Fig 5-2: “1:5” instead of “5:1” reward; same for Fig6. “1:4” instead of “4:1"

We apologize for these typos and have corrected them.

- P. 18 l. 355: For consistency, consider sticking to “more” instead of “less” and “fewer"

We now use “more” consistently throughout.

- P. 18 l. 356-357: The sentence in brackets may be distracting

We agree, and have removed this statement.

- P. 23 l. 438-440: The increasing bias to more frequent targets is hard to spot in Figure 6

We apologize, this effect was obscured by the analysis error we discovered (noted above). We

regenerated this figure after correcting that error, and it is now much easier to observe the frequency

effect by eye.

- P. 25: You present positive correlation between risk-seeking and proportion of intermediate

movements in the text but show negative correlation in Figure 8A between risk-aversion and proportion

of intermediate movements. Consider using consistent terms in the text and figure.

We apologize for this discrepancy, and have revised our statement and our figure to be more consistent.

- Fig 8: Please indicate that lower values denote risk-seeking and higher values risk-averse behavior

We apologize for the lack of clarity, and now state in the figure legend how to interpret the axes in this

figure.

- It would also be helpful to note the outlier in Fig 8

We now plot the outlier in Fig. 8, although this individual has been excluded from our regression

estimates.
